# Modeled Impact of Seasonal Malaria Chemoprevention on District-Level Suspected and Confirmed Malaria Cases in Chad Based on Routine Clinical Data (2013–2018)

**DOI:** 10.4269/ajtmh.21-0314

**Published:** 2021-10-18

**Authors:** Sol Richardson, Azoukalne Moukenet, Mahamat Saleh Issakha Diar, Monica Anna de Cola, Christian Rassi, Helen Counihan, Arantxa Roca-Feltrer

**Affiliations:** ^1^ Malaria Consortium, The Green House, London, United Kingdom;; ^2^ Malaria Consortium, Angle bureau de l’Entente des Eglises (EEMET), N’Djaména, Chad;; ^3^ Programme National de Lutte contre le Paludisme du Tchad, N’Djaména, Chad

## Abstract

Sulfadoxine-pyrimethamine plus amodiaquine is delivered to children aged 3–59 months as seasonal malaria chemoprevention (SMC) in areas where transmission is highly seasonal such as Chad and other Sahelian countries. Although clinical trials show a 75% reduction in malaria cases, evidence of SMC’s impact at scale remains limited. Using data from the Chadian National Health Management Information System, we analyzed associations between SMC implementation during July–October and monthly district-level malaria incidence (suspected and confirmed outpatient cases) among children aged 0–59 months at health facilities in 23 health districts with SMC implementation during 2013–2018. Generalized additive models were fitted with separate cyclic cubic spline terms for each district to adjust for seasonality in cases. SMC implementation in Chad was associated, compared with no implementation, with lower monthly counts of both suspected (rate ratio [RR]: 0.82, 95% CI: 0.72–0.94. *P* = 0.006) and confirmed malaria cases (RR: 0.81, 95% CI: 0.71–0.93, *P* = 0.003), representing around 20% reduction in malaria incidence. Sensitivity analyses showed effect sizes of up to 28% after modifying model assumptions. Caution should be exercised in interpreting our findings, which may not be comparable with other studies, and may over- or underestimate impact of SMC; not all malaria cases present at health facilities, not all suspected cases are tested, and not all facilities report cases consistently. This study’s approach presents a solution for employing readily available routine data to evaluate the impact of health interventions at scale without extensive covariate data. Further efforts are needed to improve the quality of routine data in Chad and elsewhere.

## INTRODUCTION

Seasonal malaria chemoprevention (SMC) through administration of sulfadoxine-pyrimethamine (SP) in combination with amodiaquine (AQ) at monthly intervals to children aged 3–59 months is recommended as an intervention against *Plasmodium falciparum* malaria during the annual high-transmission season in regions where the majority (> 60%) of clinical malaria cases occur during a period of 4 months, the clinical attack rate of malaria is greater than 0.1 attack per transmission season in the target age group, and SP plus AQ (SPAQ) remains efficacious.^1^

Seasonal malaria chemoprevention is considered a cost-effective intervention,^2^^–^^5^ with annual cost of delivery per child in Chad in 2016 estimated at US$3.86, and costs of US$10.26 and US$39.20 per malaria case and disability-adjusted life year averted among eligible children.^4^

It has also been found to be a highly efficacious intervention in terms of reduction of malaria morbidity, prevalence of malaria parasitemia, malaria-related hospital admissions, and mortality.^6^^–^^12^ A number of randomized controlled trials have been conducted to assess the protective efficacy of SMC against malaria cases in eligible children. A meta-analysis of SMC studies with monthly administration of SPAQ to children aged less than five years during the peak malaria transmission season showed an 83% (95% CI: 72–89) reduction in the incidence of clinical attacks of malaria and a similar reduction in incidence of severe malaria.^13^ Among these studies, a trial in Senegal conducted by Cisse et al.^14^ found that the prevalence ratio of *P. falciparum* parasitemia diagnosed using rapid diagnostic tests (RDT) was 68% lower (95% CI: 35–85, *P* = 0.002) among children aged 3–59 months in areas that received SMC with SPAQ over September to November than in control areas. Meanwhile, a randomized, blinded placebo-controlled study by Dicko et al.^15^ in three localities near Bamako, Mali, found that SMC using SPAQ over August to September resulted in a protective effect of 82% (95% CI: 78–85, *P* < 0.001) against clinical episodes of *P. falciparum* malaria. A trail by Konaté et al.^16^ in Burkina Faso showed similar results.

Evidence from the Unitaid-funded project Achieving Catalytic Expansion of SMC in the Sahel (ACCESS-SMC) has shown the impact of SMC at scale.^17^ Case-control studies in five countries showed that SMC treatment was associated with a protective effectiveness against clinical malaria of 88.2% (95% CI: 78.7–93.4). Meanwhile, secondary analyses based on HMIS data and health facility records using a difference-in-differences approach, in the context of around 75% coverage, found reductions in confirmed outpatient malaria cases of between 25.5% (95% CI: 6.1–40.9) and 55.2% (95% CI: 42.0–65.3) across seven countries. In Chad, there was a reduction of 43.6% (95% CI: 17.8–61.3) based on records from 11 health facilities.^17^

### Seasonal malaria chemoprevention in Chad.

In Chad, the SMC program is under the supervision of the Chadian national malaria control program (PNLP, or *Program National de Lutte contre le Paludisme du Tchad*). It is primarily delivered door-to-door over four consecutive monthly cycles spanning July to October by trained SMC community distributors.^18^ Each cycle, eligible children are administered one dispersible tablet of SP and one of AQ on the first day under the supervision of SMC distributors, and single doses of AQ on the second and third days by their primary caregivers or other family members.

Seasonal malaria chemoprevention was first delivered in Chad under the leadership of the Chadian National Malaria Control Program (NMCP) in four health districts in 2013 and in one district in 2014 with support from the United Nations Children’s Fund (UNICEF). Starting in 2015, ACCESS-SMC was launched in Chad and six other countries with the objectives of removing barriers to SMC scale-up and shaping the market for SMC medicines.^17^ In 2015, SMC was also delivered by UNICEF and the French Red Cross (*Croix-Rouge française*), and in 2016 the Global Fund (the Global Fund to Fight AIDS, Tuberculosis and Malaria) also started to support SMC implementation in Chad. After the completion of ACCESS-SMC in 2017, Malaria Consortium (the lead organization in that program) has continued to support SMC implementation using philanthropic funding. Table 1 summarizes the numbers of Chadian health districts in which SMC was implemented by year and supporting organization, eligible and total districts by year, proportions of eligible and total districts in which SMC was implemented, estimated number of eligible children living (3–59 months) in eligible districts targeted for SMC delivery by year, and yearly proportions of total eligible children nationally in eligible districts targeted for SMC during 2013–2019.

**Table 1:** Numbers of Chadian health districts in which SMC was implemented by year and supporting organization, eligible and total districts by year, and proportions of eligible and total districts in which SMC was implemented (2013–2019)

Year	Supporting organization						Districts by year and percent with SMC implementation						
	Chadian NMCP	ACCESS-SMC	Malaria Consortium	UNICEF	Global Fund	French Red Cross	Eligible districts covered	Eligible districts*	Percent eligible districts covered (%)	Total districts*	Percent total districts covered (%)	Estimated number of eligible children† targeted for SMC in eligible districts (thousands)	Percentage of total eligible children† in eligible districts targeted for SMC (%)
2013	4	0	0	0	0	0	4	39	10.3	72	5.6	186	11.3
2014	0	0	0	1	0	0	1	42	2.4	77	1.3	23	1.3
2015	0	6	0	3	0	2	11	44	25.0	81	13.6	408	23.2
2016	0	14	0	4	3	0	22	54	40.7	101	21.8	781	42.9
2017	0	14	0	0	14	0	28	58	48.3	114	24.6	979	52.0
2018	0	0	15	4	14	0	33	59	55.9	118	28.0	1,131	58.1
2019	0	0	20	4	17	0	41	61	67.2	126	32.5	1,278	63.6

ACCESS-SMC = Achieving Catalytic Expansion of Seasonal Malaria Chemoprevention in the Sahel; Global Fund = the Global Fund to Fight AIDS, Tuberculosis and Malaria; NMCP = Chadian National Malaria Control Program (*Program National de Lutte contre le Paludisme du Tchad*); SMC = seasonal malaria chemoprevention; UNICEF = United Nations Children’s Fund.

* Numbers of health districts increased over time as a result of subdivision of districts. Numbers of districts shown are based on eligible and total districts in January of each calendar year. Numbers of eligible districts in 2013 included Léré health district, which was categorized as eligible in 2013, but not from 2014 onward when it was recategorized as ineligible.

† Refers to children aged 3–59 months; numbers of eligible children were calculated based on DSSIS data and the assumption that children aged 0–2 months comprise 0.15% of children aged 0–59 months in all districts.

### Study objective.

Although the protective efficacy of SPAQ against clinical episodes of *P. falciparum* malaria has been demonstrated by controlled trials, there remains limited evidence of the effectiveness of SMC at scale at a national level in Chad and other countries despite the widespread introduction of SMC since 2013.

The objective of this study was to investigate the association between implementation of SMC in Chad during the period January 2013–December 2018, and district-level rates of suspected and confirmed outpatient malaria cases reported at primary health facilities, using readily available routine clinical malaria data, and to calculate a pooled estimate of effect for SMC across multiple districts.

## MATERIALS AND METHODS

### Setting, malaria case management, data sources, and variable definitions.

In 2013, there were 72 health districts in Chad, of which 39 were eligible for SMC. Since 2014 health districts were progressively subdivided to create additional smaller districts. SMC was implemented in at least one season in 23 of the 39 original eligible health districts during January 2013–December 2018 (inclusive). This study was conducted on the basis of 2013 health districts, with data reaggregated from subdivided health districts where applicable.

All individuals presenting at health facilities with symptoms of fever were defined as suspected malaria cases. Cases were primarily confirmed using RDT, in accordance with a national policy adopted in 2009 to provide for free RDT at all registered facilities. A minority of cases were confirmed by microscopy; (despite reduced popularity in favor of RDT); this occurred primarily at urban health facilities and private clinics that retain the capacity for microscopy, and often at patients’ own expense. Microscopy was mainly used because of the unavailability of RDT, or to confirm the results of RDT. Although diagnostic criteria were applied uniformly across all health districts, availability of RDT may have varied between districts and over time (with progressive improvement in RDT availability, particularly since 2015).

Data on health district-level monthly counts of suspected and confirmed cases were obtained from the Chadian Health Management Information System (HMIS) compiled by the Chadian Ministry of Health’s Directorate for Health Data and Health Information (DSSIS: *Direction des Statistiques Sanitaires et de l’Information Sanitaire*). In brief, health facility data on a range of indicators, including attendance for malaria by age group, are collected by health facilities and district hospitals using record books provided through the NMCP, transferred to HMIS reporting forms, and converted to electronic format by district managers. The HMIS data are sent from districts by e-mail or flash drives to provincial health delegations, and finally to the DSSIS where data are compared with monthly malaria reports produced by the NMCP (based on reporting forms submitted by health facilities) to ensure consistency of data, and compiled into a HMIS database.^19^ The HMIS reporting procedures remained the same between districts and over the study period. Data quality assurance (DQA) at the health facility level varied widely, but the same DSSIS DQA procedures were applied to all districts.

Malaria cases were reported at the district level for each month by combining numbers of monthly cases reported from all government-registered primary health facilities in that district which provided data in that month. District-level observations comprised numbers of monthly suspected malaria cases and confirmed cases (defined as suspected cases with confirmed malaria infection by either rapid diagnostic testing or microscopy, without double-counting of cases diagnosed by both methods). In addition, HMIS data included numbers of primary health facilities in each district by month, and the number of facilities that provided data to the NMCP in that month.

Data on district-level populations of children aged 0–59 months were obtained from the DSSIS.† Mid-year population estimates were available for 2019; these were projected backward based on a constant population growth rate of 3.4% to estimate district-level populations by year for 2013–2018.

### Descriptive analysis.

We calculated monthly rates of both suspected and confirmed malaria cases per 1,000 children aged 0–59 months‡ for each district over the study period. This was accomplished by dividing the numbers of cases by annual estimates of district-level population provided by DSSIS multiplied by a factor representing the proportion of health facilities which provided data on malaria cases to NMCP in that month to correct for underestimation of incidence because of nonreporting.

Tables were compiled to show eligible districts that received and did not receive SMC during the study period, in addition to ineligible districts, by estimated population of children aged 0–59 months over 2013–2019.

### Statistical analysis.

Using data from the 23 eligible 2013 districts where SMC was delivered in at least one year during the period 2013–2018, we fitted generalized additive mixed models using a quasi-Poisson link function for count outcomes to test the association between months of SMC implementation and reported malaria cases in children aged 0–59 months using R version 3.6.2 for Windows. Two models were fitted for different outcomes: suspected cases (Model 1) and confirmed cases (Model 2). Both were expressed as count measures of monthly cases by 2013 district (combining cases from districts that had been subdivided from original 2013 districts).

The primary exposure, SMC implementation, was expressed as a binary variable with “1” corresponding to the expected period of protection (July–October inclusive). In 2016, the 2013 district of Moussoro was subdivided into the districts of Chaddra, Michemire, Moussoro, and Salal. In that year, SMC was implemented in the districts of Moussoro and Chaddra only. The exposure variable was coded as “0.745,” representing the estimated proportion of the population in these subdivided districts targeted for SMC, maintaining the 2013 Moussoro boundaries.

Year and health district were fitted as random effects (with years nested within health districts), to account for contextual factors varying between districts and over time. Cyclic cubic spline terms were fitted on month using the R package mgcv individually by health district on month to adjust for seasonality of malaria cases. Models used an offset term to account for district-level population change over time and proportion of facilities reporting malaria cases. This was accomplished by log-transforming the product of the annual estimates of district-level population provided by DSSIS and the factor representing the proportion of health facilities that provided data on malaria cases in each month by district.

**Figure 1.**
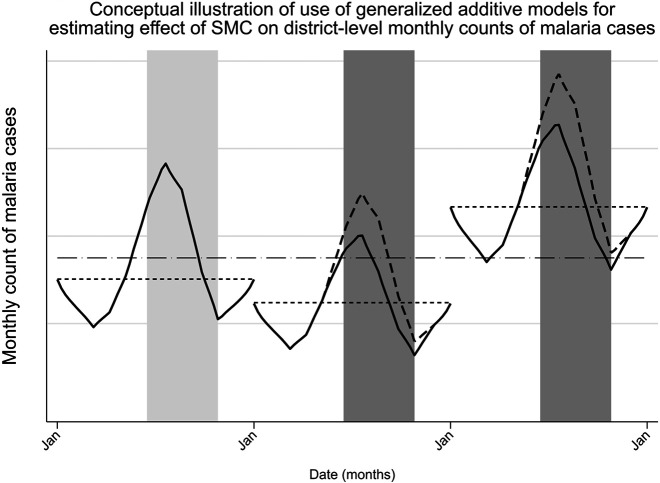
Conceptual illustration of use of generalized additive models for estimating the effect of seasonal malaria chemoprevention (SMC) on district-level monthly counts of malaria cases. Modeling approach. The graph shows hypothetical fitting of mean monthly counts of malaria cases in a district over 3 years (with SMC delivery in the latter 2 years) using generalized additive mixed models. Gray areas correspond to July–October; darker gray represents periods in which SMC was delivered. The dash-dotted line represents the district-level random intercept. Dotted lines represent random effects for each year, fitted as random intercepts and nested within the district. Differences in mean monthly malaria cases between years are illustrated by differences between dotted lines. Solid line curves show hypothetical monthly malaria case counts fitted by the model based on cyclic cubic splines (for seasonality). Curves show a hypothetical 10% reduction in rate of malaria cases (rate ratio: 0.90) during SMC delivery. Dashed line curves represent counterfactual expected rates of malaria cases without effect of SMC. The relative difference between solid and dashed line curves corresponds to the rate ratio. Effect of SMC was assumed to be uniform across all districts and periods with SMC delivery; rate ratios represent pooled estimates of effect across districts and years.

Results were expressed as rate ratios (RR) for monthly cases during months in which SMC was implemented relative to months in which it was not after seasonal adjustment, with 95% CIs. Rate ratios were used to calculate a pooled estimate of effect for SMC across the 23 districts. Estimated degrees of freedom (EDF, with a value of 1 corresponding to a linear effect) were calculated for each district’s spline term along with *P* values.^20^^,^^21^ Splines were plotted for each health district, representing monthly seasonality in malaria cases, were graphed for both Model 1 and Model 2 using the R package ggplot2. Figure 1 illustrates the modeling approach used.

We hypothesized that counts of confirmed monthly malaria cases of “0” may have been attributable to issues with testing or reporting, rather than an absence of cases given their apparent clustering in specific districts at the beginning of the study period. We therefore undertook a sensitivity analysis by refitting Model 2 for children aged 0–59 months with the assumption that monthly observations of “0” were missing data.§

The analysis assumed that SMC coverage was 100% across all districts, and district-level counts of malaria cases included children aged 0–2 months who were not targeted for SMC. We therefore performed sensitivity analyses by refitting Models 1 and 2 with assumed coverages of SMC of 80% and 90%, and restricting our outcome measure to cases among children aged 12–59 months.‖

## RESULTS

### Descriptive analysis.

Health districts were subdivided from 72 in 2013 to 118 in 2018 (of which 59 were eligible and 59 noneligible). Based on the DSSIS data, it was estimated that the number of children aged 3–59 months targeted to receive SMC increased from 186,000 in 2013 to just over 1.13 million by 2018 as implementers expanded their programs to new districts (Table 1).

**Figure 2.**
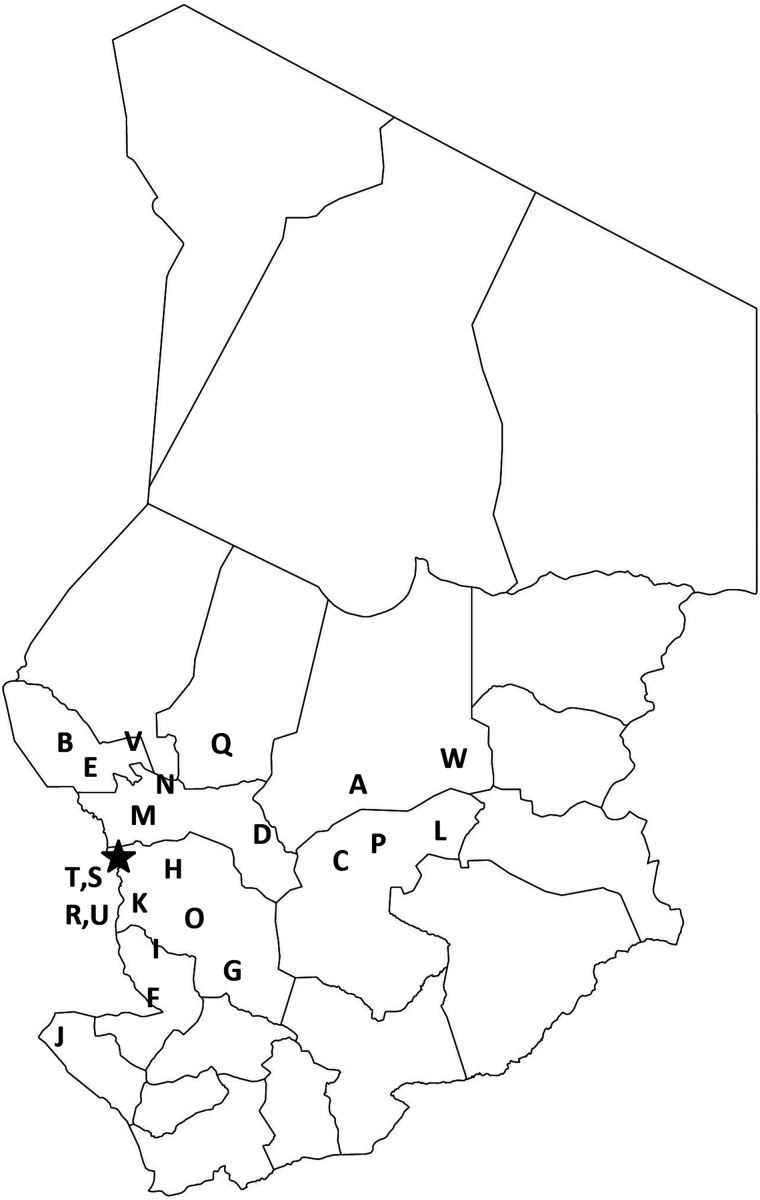
Map of regions of Chad with locations of eligible health districts in which seasonal malaria chemoprevention (SMC) was implemented during 2013–2018. The following letters represent 2013 districts that received SMC during the period 2013–2018: A: Ati; B: Bagassola; C: Bitkine; D: Bokoro; E: Bol; F: Bongor; G: Bousso; H: Dourbali; I: Guelendeng; J: Léré; K: Mandelia; L: Mangalmé; M: Massaguet; N: Massakory; O: Massenya; P: Mongo; Q: Moussoro; R: N’Djaména Center; S: N’Djaména Est; T: N’Djaména Nord; U: N’Djaména Sud; V: Ngouri; W: Oum Hadjer.

**Figure 3.**
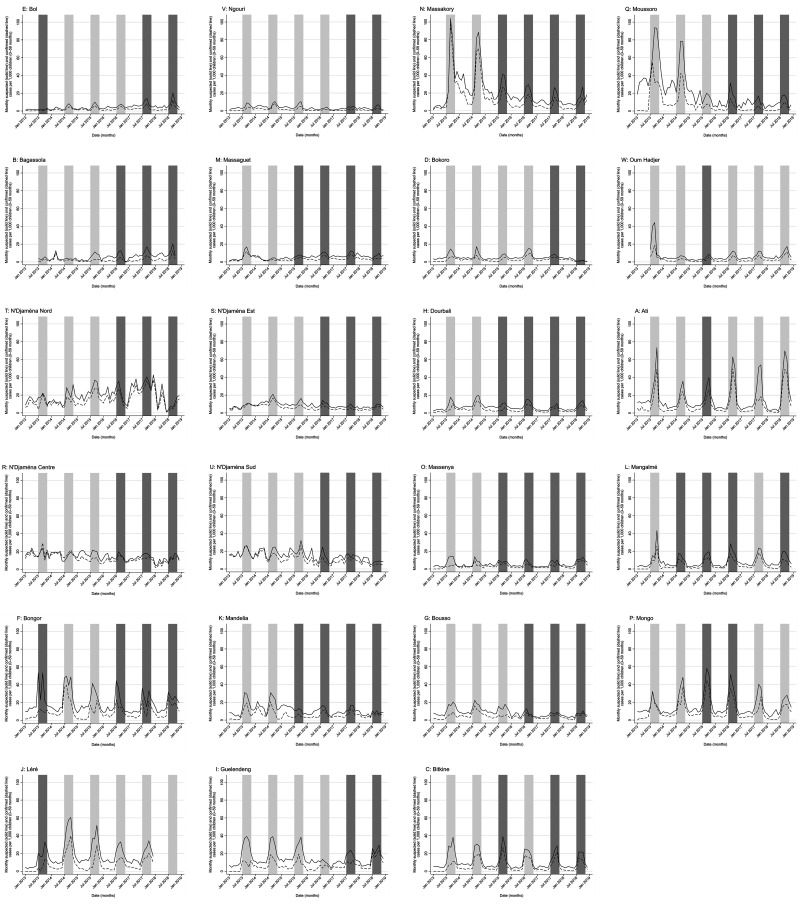
Numbers of monthly suspected (solid line) and confirmed (dashed line) malaria cases among children aged 0–59 months in primary health facilities, and periods of seasonal malaria chemoprevention (SMC) rounds (gray areas), in 23 Chadian health districts in which SMC was implemented during 2013–2018. Gray areas correspond to the period of SMC delivery and duration of effect (July–October); darker gray represents periods in years in which SMC was delivered in each district, whereas lighter areas represent the high-transmission season in years without SMC delivery for comparison. Rates of suspected and confirmed cases in Moussoro district from January to December 2013 calculated on assumption of case reporting from 100% of health facilities.

Figure 2 shows a map of Chad, with locations of all 23 health districts where at least one round of SMC implementation occurred during 2013–2018 marked. In 2013, these districts had an estimated 1.11 million children aged 0–59 months. Figure 3 displays monthly suspected and confirmed malaria cases among children aged 0–59 months reported at primary health facilities by district over the study period as a rate per 1,000 children of that age. The months in which SMC took place in each district are shown in gray. Of the 899,115 suspected malaria cases in recorded among children aged 0–59 months in the 23 districts during the study period, 526,711 (58.6%) were confirmed by RDT or microscopy. The proportion of suspected cases confirmed remained similar throughout the study period.

On average, 89.5% (95% CI: 88.9–90.1) of health facilities across all 23 districts provided data on malaria cases to the NMCP across all months of the study period. Data on monthly suspected and confirmed malaria cases during the periods January–June 2013 and October–December 2018 (inclusive) were missing for Bagassola district, and January–June 2013 for Oum Hadjer, whereas no data were available for Léré for 2018. Data on proportions of facilities reporting malaria cases to HMIS were missing for Moussoro for 2013. Data from these districts and months were not included in the analytic dataset.

Supplemental Tables 1–3 show estimated populations of children aged 0–59 months by year for each 2013 district based on DSSIS data over the period 2013–2019, for eligible districts that received SMC during 2013–2018 (Supplemental Table 1), eligible districts that did not receive SMC (Supplemental Table 2), and ineligible districts (Supplemental Table 3). All tables show the evolution of health districts as they divided over time to create new districts. Table S1 displays the years in which eligible districts received SMC and organizations supporting implementation by year.

### Statistical analysis.

The results of the statistical analyses show that district-level rates of reported suspected (Model 1 RR: 0.82, 95% CI: 0.72–0.94. *P* = 0.006) and confirmed (Model 2 RR: 0.81, 95% CI: 0.71–0.93, *P* = 0.003) malaria cases were significantly lower during the months of the high-transmission season in years in which SMC was implemented than in years without SMC (Table 2). These results indicate a reduction in expected monthly cases of just under 20% during periods of SMC implementation.

**Table 2:** Results of generalized additive mixed models for associations between periods implementation of SMC and rates of suspected and confirmed malaria cases in 23 health districts

Parameter	Model 1 (suspected cases)		Model 2 (confirmed cases: RDT or microscopy)	
SMC implementation	RR (95% CI)	*P* value	RR (95% CI)	*P* value
Month SMC implemented*	0.82 (0.72–0.94)	0.006	0.81 (0.71–0.93)	0.003
District-level spline terms	EDF†	*P* value	EDF	*P* value
A: Ati	3.98	< 0.001	3.59	< 0.001
B: Bagassola	3.04	< 0.001	3.11	0.137
C: Bitkine	4.47	< 0.001	3.45	< 0.001
D: Bokoro	3.78	< 0.001	3.09	< 0.001
E: Bol	3.57	0.030	3.61	< 0.001
F: Bongor	6.56	< 0.001	6.43	< 0.001
G: Bousso	2.45	0.024	2.59	0.004
H: Dourbali	2.96	0.008	2.70	0.002
I: Guelendeng	3.38	< 0.001	3.02	< 0.001
J: Léré	5.28	< 0.001	5.66	< 0.001
K: Mandelia	2.14	0.450	3.24	0.002
L: Mangalamé	2.96	< 0.001	2.84	< 0.001
M: Massaguet	2.92	0.057	3.22	0.011
N: Massakory	4.66	< 0.001	4.71	< 0.001
O: Massenya	2.43	0.008	2.28	0.004
P: Mongo	4.43	< 0.001	4.39	< 0.001
Q: Moussoro	0.75	0.702	1.50	0.396
R: N’Djaména Center	3.71	0.298	3.83	0.011
S: N'Djaména Est	3.13	0.074	3.32	0.011
T: N’Djaména Nord	2.80	0.293	3.17	0.039
U: N’Djaména Sud	4.08	0.054	4.10	0.005
V: Ngouri	3.77	0.024	3.32	0.004
W: Oum Hadjer	3.02	0.001	2.61	0.000

EDF = estimated degrees of freedom; RR = rate ratio; SMC = seasonal malaria chemoprevention.

* Corresponds to the association between the implementation of SMC in a given month and suspected and confirmed cases in the same month, relative to the expected number of cases if SMC had not been implemented (predicted based on seasonality of malaria incidence in years without SMC implementation), with effect sizes expressed as rate ratios for monthly cases.

† The EDF is a measure of how “wiggly” the smooth term is (i.e., EDF = 1 corresponds to a linear effect). The EDF can be considered the equivalent to the polynomial order of the smooth term plus 1. The *P* value is used to measure the statistical significance of the smooth term’s difference from a linear effect.

**Figure 4.**
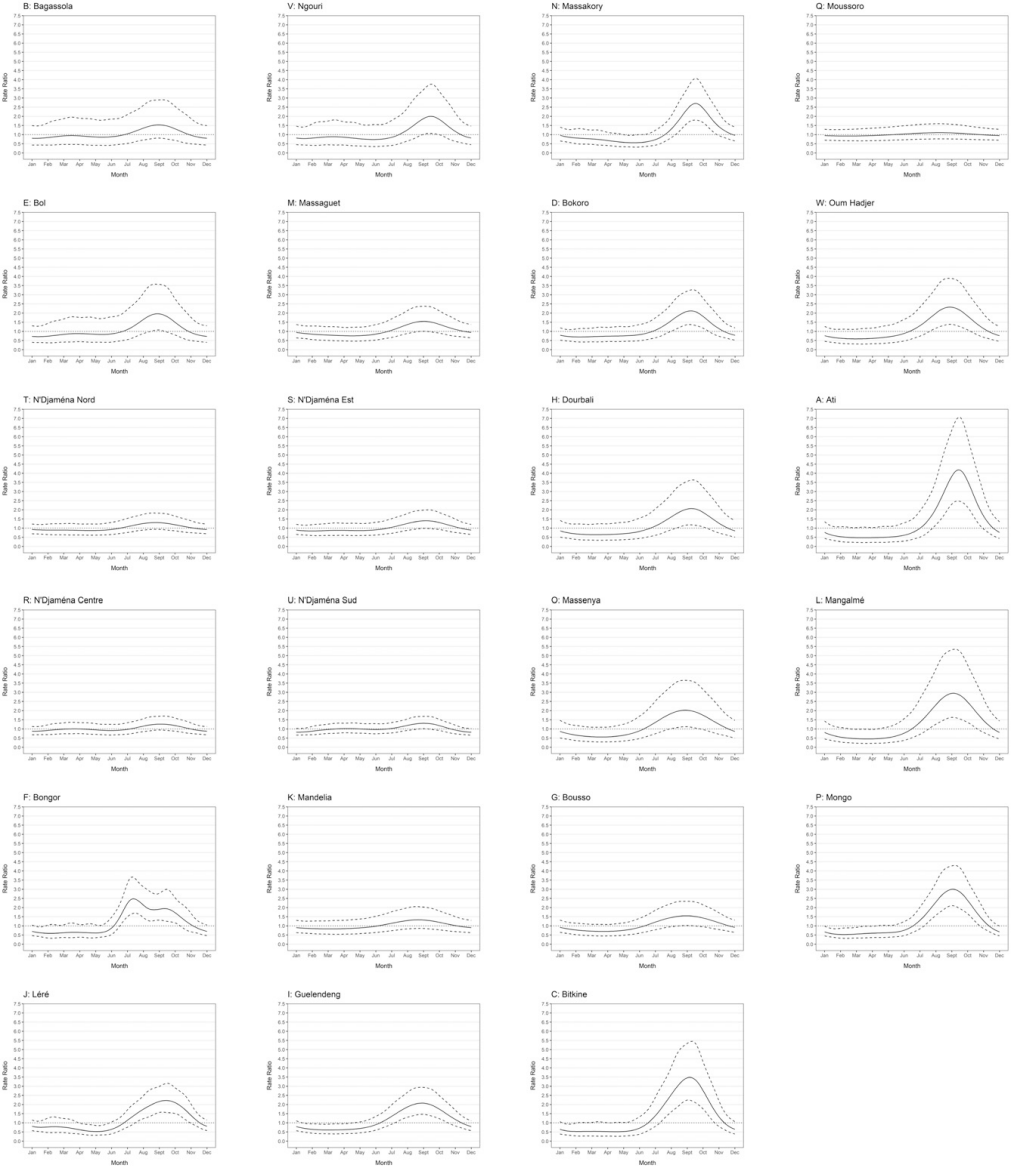
Graphs of cyclic cubic spline terms fitted for seasonality in monthly district-level counts of suspected malaria cases among children aged 0–59 months in primary health facilities (Model 1) in 23 Chadian health districts in which seasonal malaria chemoprevention (SMC) was implemented during 2013–2018. Graphs show model-fitted cyclic cubic spline terms (solid line), representing predicted rates of suspected malaria cases over January–December as a ratio relative to the annual grand monthly mean (dotted line), with 95% CI (dashed lines).

Figure 4 and Supplemental Figure 1 show graphs of cubic spline terms by district, used to adjust for seasonality in district-level monthly counts of suspected and confirmed malaria cases among children aged 0–59 months, as predicted by Models 1 and 2. The curves represent relative predicted numbers of confirmed cases per month compared with the grand mean for the year, expressed RRs, and are displayed with 95% CIs.

There were reported counts of “0” confirmed cases for 33 months across eight districts, of which 31 occurred in 2013. The results of the sensitivity analysis show that the estimate of effect of SMC implementation was unchanged after excluding months in which counts of confirmed cases among children aged 0–59 months were recorded as “0” in HMIS (RR: 0.81, 95% CI: 0.71–0.93, *P* = 0.003). We found a greater size of effect for SMC implementation on suspected (RR: 0.78, 95% CI: 0.67–0.93, *P* = 0.006) and confirmed cases (RR: 0.77, 95% CI: 0.65–0.91, *P* = 0.003) among children aged 0–59 months when coverage was assumed to be 80%.

Effect sizes were greater after refitting models for observations of monthly counts of suspected and confirmed cases among children aged 12–59 months, and greater still after varying assumed SMC coverage (Table 3); for example, at 80% assumed coverage, we found an effect size of 28% (RR: 0.72, 95% CI: 0.61–0.84, *P* < 0.0001).

**Table 3:** Results of sensitivity analyses for associations between periods implementation of SMC and rates of suspected and confirmed malaria cases in 23 health districts, among children aged 0–59 months and 12–59 months, at different assumed levels of SMC coverage

Parameter	Model 1 (suspected cases)		Model 2 (confirmed cases: RDT or microscopy)	
Children age 0–59 months, assumed coverage*	RR (95% CI)	*P* value	RR (95% CI)	*P* value
100%	0.82 (0.72–0.94)	0.006	0.81 (0.71–0.93)	0.003
90%	0.80 (0.69*–*0.94)	0.006	0.79 (0.68*–*0.92)	0.003
80%	0.78 (0.67*–*0.93)	0.006	0.77 (0.65*–*0.91)	0.003
Children age 12–59 months, assumed coverage*	RR (95% CI)	*P* value	RR (95% CI)	*P* value
100%	0.77 (0.67*–*0.87)	< 0.0001	0.78 (0.68*–*0.90)	< 0.001
90%	0.74 (0.64–0.85)	< 0.0001	0.76 (0.65*–*0.89)	< 0.001
80%	0.72 (0.61*–*0.84)	< 0.0001	0.73 (0.62*–*0.88)	< 0.001

RDT = rapid diagnostic test; RR = rate ratio; SMC = seasonal malaria chemoprevention.

* Corresponds to the association between administration of SMC in a given month at a given assumed level of coverage (of the respective age group), and suspected and confirmed cases in the same month, relative to the expected number of cases if SMC had not been administered, with effect sizes expressed as rate ratios for monthly cases.

## DISCUSSION

Although not the first to evaluate impact malaria interventions using HMIS systems,^22^ or of SMC in Chad,^17^ this study was able to do so at a country-wide scale covering 23 health districts (with 496 health facilities in 2016). Our results showed an 18% reduction in suspected cases, a 19% reduction in confirmed cases reported at primary health facilities among children aged 0–59 months at the district-level during the months of SMC implementation. However, this estimate of SMC’s impact on malaria is substantially below the expected reduction in clinical malaria episodes of around 75%,^6^ or that found in previous trial-based studies.^13^^–^^16^ Previous findings on the impact of SMC vary by study type, even across the same settings; the evaluation of ACCESS-SMC found a reduction of 42.4% in malaria incidence across six countries based on HMIS data, whereas case-control studies using individual-level data across five of the same countries found a pooled effect size of 88.2%.^17^

Our estimate of protective efficacy, based on a secondary analysis of routine data to evaluate SMC implementation at scale, may not be comparable with previous studies for various reasons. First, impact is dependent on coverage among eligible children, which may have varied between health districts and over time. Although covariate adjustment for coverage was not possible because of lack of comprehensive data for 2013–2018, a SMC end-of-round survey in 2020 found that coverage of SPAQ was over 90% of eligible children across all four cycles, and caregivers’ adherence to administration of AQ in days following distributor visits was over 97% across all cycles, across 20 districts where SMC was supported by Malaria Consortium.^23^ Second, timing of SMC cycles may not have corresponded exactly with July–October (assumed to be the period of effect of SMC for the purposes of modeling); timing of cycles and intervals between them may have been inconsistent between districts, and between years (e.g., because of delays or stock-outs of commodities). Third, analyses of cases among children 0–59 months may have underestimated effect sizes for SMC implementation as children 0–2 months were not targeted for SMC but cases in this group were included in the outcome measure; it should be noted, however, that a very small proportion of pediatric malaria cases occur in this age group in African settings.^24^

The results of our sensitivity analyses showed larger effect sizes with assumed SMC coverage of under 100%, and refitting models for counts of cases among children aged 12–59 months. Although CIs of all model RRs overlapped, these findings highlight that estimates of SMC effectiveness at scale are sensitive to model assumptions.

### Strengths and limitations.

HMIS systems are a rich and readily available source of data for evaluation of public health interventions.^25^ In the absence of usable covariate data covering all eligible health districts and months of the study period, for example, relating to climate†† or population demographics, the use of spline terms and random effects to account for seasonality (i.e., adjusting for differences in district-level malaria incidence between high- and low-transmission seasons) and differences in underlying risk of malaria transmission between districts and years presented a method to adjust for contextual factors influencing malaria incidence at the district level for which sufficient data were not available.

Data were insufficient to adjust for effects of other interventions as fixed effects and only available for 2014–2015 from a Demographic and Health Survey,^26^ which found that, in the 23 districts studied, household ownership of at least one long-lasting insecticidal bed net ranged from 53.8% in the region of Barh El Gazel (Moussoro district) to 90.1% in Mayo Kebbi Ouest (Léré district), whereas coverage with indoor residual spraying within the past 12 months was 2.2% (N’Djamena) or less. It was not possible to determine whether coverage of these interventions varied over time, or by district within regions; use of random intercepts for district and year implicitly adjusted for this variation, however.

Use of HMIS data for impact evaluations raises issues over internal validity, completeness, and potential bias in estimates of effect,^23^ and caution must be exercised in interpreting their findings.^27^ There are three major limitations relating to the use of passive malaria surveillance systems such as HMIS: 1) only a fraction of infected individual cases seek treatment for malaria at (government-registered) health facilities; 2) of those patients who seek care, not all are tested using parasitological diagnosis; and 3) not all facilities report malaria cases consistently over time.^28^ One study, based on data from 2007, found that less than 10% of children aged 0–59 months with fever attended a health facility;^29^ this suggests that there is considerable underreporting of malaria cases in HMIS. Derivation of variables representing stockouts of RDTs or proportions of suspected cases tested was not possible with available data. In addition, microscopy was used to a different extent across districts, and in some may have represented a fall-back method in case of RDT stockouts. Diagnostic methods vary in sensitivity and specificity, with implications for accuracy of reporting of confirmed cases.^30^ In addition to reports of data quality issues in HMIS by the Chadian Ministry of Health,^31^ a recent audit of quality of HMIS data by Moukénet et al.^32^ including all government-registered health facilities in the district of Massaguet showed overreporting of both suspected and confirmed malaria cases among children aged 1–4 years by a factor of more than two in the HMIS database during the high-transmission season compared with records in logbooks obtained directly from health facilities. Other studies in comparable settings report substantial overreporting of confirmed malaria cases, misdiagnosis, and overprescription of anti-malarials.^33^^,^^34^ Although this study was able to detect a significant effect for SMC on malaria incidence despite data quality issues (likely as a result of the relatively large effect size outweighing inconsistencies in case reporting), further efforts are required to improve routine clinical data and its reporting in Chad and other African settings,^35^ account for data quality issues when evaluating impacts of health interventions, and define quality criteria for inclusion of data in such evaluations.

One limitation of the exposure variable was that SMC coverage in each model was assumed to be the same across districts and remain constant between years. It was not possible to adjust for actual coverage as data on district-level coverage were not available for the study period.

Use of annual district-level population estimates based on 2019 DSSIS data and with back projection using a constant growth rate to calculate incidence of malaria cases and generate model offset terms had several limitations. Accuracy of district-level populations by year was dependent on that of DSSIS estimates. The actual growth rate of the population of children aged 0–59 months may not have matched our assumption of 3.4% over time and across districts. Although it did not account for monthly changes in population, or movements by migratory or nomadic populations,^36^ to our knowledge there were no large population movements in the districts analyzed during the study period.

During the study period, there were a few instances of transfers of health facilities between districts; for example, one health facility was transferred from Dourbali to N’Djaména Sud. Although this was reflected in recorded numbers of facilities by district, accuracy of district-level population estimates would have been undermined. This is likely to have had only a negligible effect on model results, however.

Estimates of monthly incidence of suspected and confirmed malaria cases among children aged 0–59 months, and generation of model offset terms, also accounted for the proportion of health facilities in each district reporting malaria cases to HMIS. Although populations of individual facility catchment areas were available in DSSIS population data, it was not possible to weight facilities by catchment population when calculating malaria incidence and generating model offset terms as the names of facilities not reporting malaria cases to the NMCP were not shown in HMIS. Our analysis was therefore performed on the assumption that each facility represented an equal proportion of that district’s population. This assumption, however, may have led to bias in our descriptive and statistical analyses; for example, if smaller health facilities had a greater propensity to not report malaria data to HMIS this may have biased estimates of malaria incidence upward.

## CONCLUSION

The results of this study, based on routine HMIS data, provide evidence to support the effectiveness of SMC delivered at scale in Chad during 2013–2018 and present a viable method for evaluating public health interventions in the absence of trial studies, household surveys, and extensive covariate data. Although HMIS data is readily available, its use is subject to a number of limitations. Further studies on effectiveness of SMC at scale will use data obtained from a wider range of sources.^37^

Analyses applying the methods proposed by this study to data on monthly malaria cases aggregated at the facility level in other countries where SMC programs are delivered may allow more robust analyses to estimate the effectiveness of SMC, adjustment for facility-level variables, and consideration of facility-level data quality (i.e., following data quality audits). Use of larger datasets with cases aggregated at the facility level data may overcome the limitations associated with aggregation at the district level and facilitate secondary analyses such as testing of SMC’s duration of effect after the final cycle.

## Supplemental Material


Supplemental materials

## Footnotes

§ Data were considered missing and omitted from the primary analysis where either 1) the number of health facilities reporting cases in a given month was recorded as “0” in the HMIS database; or 2) cells showing numbers of health facilities reporting cases, or monthly numbers of cases, were left blank. In some instances, however, a number of reporting health facilities was given but number of cases was reported as “0”; these observations were omitted for the purposes of the sensitivity analysis. Observations of monthly counts of confirmed malaria cases of “0” during January–July 2013 (inclusive) occurred in eight districts, including Bitkine, Guelendeng, Léré and Mangalmé (6 months), Ngouri (3 months), Massenya and Moussoro (2 months), and Mongo (1 month). Counts of “0” confirmed cases were found in Bagassola during 2016; for the purposes of this sensitivity analysis this was assumed to be missing data.

†† Although we attempted to obtain climate data covering the entire study period for districts where SMC was implemented, including precipitation, mean minimum and maximum daily temperature, and other variables, from the Chadian National Meteorological Agency (*Direction des Ressources en Eau et de la Météorologie*), usable climate data covering 2013–2018 were only available for N’Djamena. We therefore did not consider use of climate data for the present study.

† According to DSSIS estimates, the population of children aged 0–59 months was assumed to comprise 19.93% of the total population for each district. Children aged 0–11 months were assumed to make up 4.42% of the total.

‡ In clinic registers and HMIS data, malaria cases were recorded for the age categories 0–11 months, 12–59 months, 5–14 years, and 15 years and above. Monthly counts of cases among children aged 0–59 months were calculated by summing the former two categories.

‖ Incidence of malaria among children aged 12–59 months was reported separately in HMID data, and extracted and used as the outcome variable in this sensitivity analysis. Model offset terms were recalculated based on DSSIS estimates of district-level populations for children aged 12–59 months as appropriate.

## References

[1] WHO , 2012. *WHO Policy Recommendation: Seasonal Malaria Chemoprevention (SMC) for Plasmodium falciparum Malaria Control in Highly Seasonal Transmission Areas of the Sahel Sub-region in Africa*. Geneva, Switzerland: World Health Organization.

[2] Givwell , 2018. *Malaria Consortium—Seasonal Malaria Chemoprevention – November 2018 Version.* Available at: https://www.givewell.org/charities/malaria-consortium/November-2018-version/. Accessed February 17, 2021.

[3] UNICEF Supply Division , 2017. *Seasonal Malaria Chemoprevention: Supply and Demand Update*. New York, NY: United Nations International Children’s Emergency Fund.

[4] Scott N Hussain SA Martin-Hughes R Fowkes FJI Kerr CC Pearson R Kedziora DJ Killedar M Stuart RM Wilson DP, 2017. Maximizing the impact of malaria funding through allocative efficiency: using the right interventions in the right locations. Malar J 16: 368. 28899373 10.1186/s12936-017-2019-1 PMC5596957

[5] Gilmartin C Nonvignon J Cairns M Milligan P Bocoum F Winskill P Moroso D Collins D, 2021. Seasonal malaria chemoprevention in the Sahel subregion of Africa: a cost-effectiveness and cost-savings analysis. Lancet Glob Health 9: e199–e208. 33482140 10.1016/S2214-109X(20)30475-7

[6] WHO , 2013. *Seasonal Malaria Chemoprevention with Sulfadoxine–Pyrimethamine Plus Amodiaquine in Children: A Field Guide*. Geneva, Switzerland: World Health Organization. Available at: https://apps.who.int/iris/bitstream/handle/10665/85726/9789241504737_eng.pdf?sequence=1/. Accessed February 17, 2021.

[7] Druetz T Corneau-Tremblay N Millogo T Kouanda S Ly A Bicaba A Haddad S, 2018. Impact evaluation of seasonal malaria chemoprevention under routine program implementation: a quasi-experimental study in Burkina Faso. Am J Trop Med Hyg 98: 524–533. 29260654 10.4269/ajtmh.17-0599 PMC5929206

[8] Cissé B , 2016. Effectiveness of seasonal malaria chemoprevention in children under ten years of age in Senegal: a stepped-wedge cluster-randomised trial. PLoS Med 13: e1002175. 27875528 10.1371/journal.pmed.1002175 PMC5119693

[9] Zongo I Milligan P Compaore YD Some AF Greenwood B Tarning J Rosenthal PJ Sutherland C Nosten F Ouedraogo JB, 2015. Randomized noninferiority trial of dihydroartemisinin-piperaquine compared with sulfadoxine-pyrimethamine plus amodiaquine for seasonal malaria chemoprevention in Burkina Faso. Antimicrob Agents Chemother 59: 4387–4396. 25918149 10.1128/AAC.04923-14 PMC4505196

[10] Meremikwu MM Donegan S Sinclair D Esu E Oringanje C, 2012. Intermittent preventive treatment for malaria in children living in areas with seasonal transmission. Cochrane Database Syst Rev 2012: CD003756. 10.1002/14651858.CD003756.pub4 PMC6532713 22336792

[11] Issiaka D Barry A Traore T Diarra B Cook D Keita M Sagara I Duffy P Fried M icko A, 2020. Impact of seasonal malaria chemoprevention on hospital admissions and mortality in children under 5 years of age in Ouelessebougou, Mali. Malar J 19: 103. 32126989 10.1186/s12936-020-03175-y PMC7055064

[12] Diawara F , 2017. Measuring the impact of seasonal malaria chemoprevention as part of routine malaria control in Kita, Mali. Malar J 16: 325. 28797263 10.1186/s12936-017-1974-x PMC5553795

[13] Wilson AL Tc Taskforce IP, 2011. A systematic review and meta-analysis of the efficacy and safety of intermittent preventive treatment of malaria in children (IPTc). PLoS One 6: e16976. 21340029 10.1371/journal.pone.0016976 PMC3038871

[14] Cissé B , 2016. Effectiveness of seasonal malaria chemoprevention in children under 10 years of age in Senegal: a stepped wedge cluster-randomised trial. PLoS Med 13: e1002175. 27875528 10.1371/journal.pmed.1002175 PMC5119693

[15] Dicko A , 2011. Intermittent preventive treatment of malaria provides substantial protection against malaria in children already protected by an insecticide-treated bednet in Mali: a randomised, double-blind, placebo-controlled trial. PLoS Med 8: e1000407. 21304923 10.1371/journal.pmed.1000407 PMC3032550

[16] Konaté AT , 2011. Intermittent preventive treatment of malaria provides substantial protection against malaria in children already protected by an insecticide‐treated bednet in Burkina Faso: a randomised, double‐blind, placebo‐controlled trial. PLoS Med 8: e1000408. 21304925 10.1371/journal.pmed.1000408 PMC3032552

[17] ACCESS-SMC Partnership , 2020. Effectiveness of seasonal malaria chemoprevention at scale in west and central Africa: an observational study. Lancet 396: 1829–1840. 33278936 10.1016/S0140-6736(20)32227-3 PMC7718580

[18] Malaria Consortium , 2020. *Our SMC Programme*. Available at: https://www.malariaconsortium.org/pages/seasonal-malaria-chemoprevention/our-smc-programme.htm/. Accessed February 17, 2021.

[19] PNLP , 2019. *Rapport Annuel d’activités 2018*. N’Djaména, Chad: Programme National de Lutte contre le Paludisme du Tchad.

[20] Sullivan KJ Shadish WR Steiner PM, 2014. An introduction to modeling longitudinal data with generalized additive models: applications to single-case designs. Psychol Methods 20: 26–42. 24885341 10.1037/met0000020

[21] Wood SN, 2013. On p-values for smooth components of an extended generalized additive model. Biometrika 100: 221–228.

[22] Ashton RA , 2019. Use of routine health information system data to evaluate impact of malaria control interventions in Zanzibar, Tanzania from 2000 to 2015. EClinicalMedicine 12: 11–19. 31388659 10.1016/j.eclinm.2019.05.011 PMC6677660

[23] Richardson S de Cola MA Marasciulo M Moukenet A Ibinaiye T Roca-Feltrer A Sawadogo B Rassi C, 2020. *2019 Coverage Report: Seasonal Malaria Chemoprevention in Burkina Faso, Chad and Nigeria*. London, UK: Malaria Consortium.

[24] Larru B Molyneux E ter Kuile FO Taylor T Molyneux M Terlouw DJ, 2009. Malaria in infants below six months of age: retrospective surveillance of hospital admission records in Blantyre, Malawi. Malar J 8: 310. 20038299 10.1186/1475-2875-8-310 PMC2805692

[25] Ashton RA Bennett A Yukich J Bhattarai A Keating J Eisele TP, 2017. Methodological considerations for use of routine health information system data to evaluate malaria program impact in an era of declining malaria transmission. Am J Trop Med Hyg 97: 46–57. 28990915 10.4269/ajtmh.16-0734 PMC5619932

[26] Institut National de la Statistique des Études Économiques et Démographiques (INSEED/Tchad), Ministère de la Santé Publique (MSP/Tchad), ICF International , 2014–2015. *Enquête Démographique et de Santé et à Indicateurs Multiples (EDS-MICS 2014–2015)*. Rockville, MD: INSEED, MSP and ICF International.

[27] Rowe AK Kachur SP Yoon SS Lynch M Slutsker L Steketee RW, 2009. Caution is required when using health facility-based data to evaluate the health impact of malaria control efforts in Africa. Malar J 8: 1–3. 19728880 10.1186/1475-2875-8-209 PMC2743707

[28] WHO , 2018. *Analysis and Use of Health Facility Data: Guidance for Malaria Programme Managers*. Geneva, Switzerland: World Health Organization.

[29] Gething PW Kirui VC Alegana VA Okiro EA Noor AM Snow RW, 2010. Estimating the number of paediatric fevers associated with malaria infection presenting to Africa’s public health sector in 2007. PLoS Med 7: e1000301. 20625548 10.1371/journal.pmed.1000301 PMC2897768

[30] Mfuh K Achonduh-Atijegbe OA Bekindaka ON Esemu LF Mbacop CD Ghandi K Leke RGF Taylor DW Nerurkar VR, 2019. A comparison of thick-film microscopy, rapid diagnostic test, and polymerase chain reaction for accurate diagnosis of *Plasmodium falciparum* malaria. Malar J 18: 73. 30866947 10.1186/s12936-019-2711-4 PMC6416847

[31] Ministère de la Sante Publique , 2019. *Plan National de Developpement Sanitaire 2018–2021*. N’Djamena, Chad: Ministère de la Santé Publique.

[32] Moukénet A de Cola M Ward C Beakgoubé H Baker K Donovan L Laoukolé J Richardson S, 2021. Health Management Information System (HMIS) data accuracy and factors associated in Massaguet district, Chad (article under review). 10.1186/s12911-021-01684-7 PMC8609810 34809622

[33] Afrane YA Zhou G Githeko AK Yan G, 2013. Utility of health facility-based malaria data for malaria surveillance. PLoS One 8: e54305. 23418427 10.1371/journal.pone.0054305 PMC3572108

[34] Leslie T Mikhail A Mayan I Anwar M Bakhtash S Nader M Chandler C Whitty CJ Rowland M, 2012. Overdiagnosis and mistreatment of malaria among febrile patients at primary healthcare level in Afghanistan: observational study. BMJ 345: e4389. 22833603 10.1136/bmj.e4389 PMC3404186

[35] Braa J Sahay S, 2012. Improving quality and use of data through data-use workshops: Zanzibar, United Republic of Tanzania. Bull World Health Organ 90: 379–384. 22589572 10.2471/BLT.11.099580 PMC3341693

[36] Randall S, 2015. Where have all the nomads gone? Fifty years of statistical and demographic invisibilities of African mobile pastoralists. Pastoralism 5: 22.

[37] Wharton-Smith A , 2021. Protocol for a hybrid effectiveness-implementation study to assess the feasibility, acceptability and protective effect of implementing seasonal malaria chemoprevention in Nampula province, Mozambique. JMIR Res Protoc 27855: (in press). 10.2196/27855 PMC8482168 34524109

