# Supplementary material for: Modeled Impact of Seasonal Malaria Chemoprevention on District-Level Suspected and Confirmed Malaria Cases in Chad Based on Routine Clinical Data (2013–2018)
Source: Am J Trop Med Hyg. 2021 Oct 18;105(6):1712–21. doi: 10.4269/ajtmh.21-0314 (PMC8641328; doi:10.4269/ajtmh.21-0314)
Supplement: Supplementary file 1 [file tpmd210314.SD1.pdf]

## Online supplement

**Supplemental Table S1. Summary of regions of Chad and eligible health districts by year (2013, 2018 and 2019) which received SMC (2013–2018)**

| Region | Health District† | | | Population projection (age 0–59 months), 2013 health districts | | | | | | | 2019 districts | Years of SMC implementation |
|---|---|---|---|---|---|---|---|---|---|---|---|---|
| 2013 | 2013 | 2018 | 2019 | 2013 | 2014 | 2015 | 2016 | 2017 | 2018 | 2019 | | |
| Barh El Gazel | Q: Moussoro | Chaddra (2014) | Chaddra | 60012 | 62053 | 64163 | 66344 | 68600 | 70932 | 73344 | 18211 | 2016* 2017, 2018 (G) |
| | | Michemire (2016) | Michemire | | | | | | | | 9234 | |
| | | Moussoro | Moussoro | | | | | | | | 36430 | |
| | | Salal (2016) | Salal | | | | | | | | 9469 | |
| Batha | A: Ati | Alifa (2016) | Alifa | 52750 | 54543 | 56398 | 58315 | 60298 | 62348 | 64468 | 23219 | 2015 (C) |
| | | Ati | Ati | | | | | | | | 12564 | |
| | | | Djedda (2019) | | | | | | | | 11139 | |
| | | | Koundjourou (2019) | | | | | | | | 17546 | |
| | W: Oum-Hadjer | Assinet (2016) | Assinet | 41745 | 43165 | 44632 | 46150 | 47719 | 49341 | 51019 | 19539 | 2015 (C) |
| | | Oum-Hadjer | Oum-Hadjer | | | | | | | | 31480 | |
| Chari Baguirmi | G: Bousso | Bailli (2016) | Bailli | 49257 | 50932 | 52664 | 54454 | 56306 | 58220 | 60200 | 26876 | 2016, 2017, 2018 (M) |
| | | Bousso | Bousso | | | | | | | | 24376 | |
| | | Kouno (2016) | Kouno | | | | | | | | 8947 | |
| | H: Dourbali | Dourbali | Dourbali | 30899 | 31949 | 33036 | 34159 | 35320 | 36521 | 37763 | 37763 | 2015 (A); 2016, 2017, 2018 (M) |
| | K: Mandelia | Mandelia | Mandelia | 29966 | 30985 | 32039 | 33128 | 34254 | 35419 | 36623 | 36623 | 2015 (A); 2016, 2017, 2018 (M) |
| | O: Massenya | Massenya | Massenya | 29062 | 30050 | 31071 | 32128 | 33220 | 34350 | 35518 | 35518 | 2015 (A); 2016, 2017, 2018 (M) |
| Guera | C: Bitkine | Bitkine | Bitkine | 39527 | 40871 | 42261 | 43698 | 45184 | 46720 | 48308 | 48308 | 2015, 2016, 2018 (U) |
| | L: Mangalamé | Mangalamé | Mangalamé | 22223 | 22979 | 23760 | 24568 | 25403 | 26267 | 27160 | 27160 | 2014, 2015, 2016, 2018 (U) |
| | P: Mongo | Baro (2016) | Baro | 40476 | 41852 | 43275 | 44747 | 46268 | 47841 | 49468 | 12939 | 2013 (S); 2015, 2016, 2018 (U) |
| | | Mongo | Mongo | | | | | | | | 36529 | |
| Hadjer Lamis | D: Bokoro | Bokoro | Bokoro | 81102 | 83859 | 86711 | 89659 | 92707 | 95859 | 99118 | 56178 | 2017, 2018 (G) |

| | | | | | | | | | | | |
|---|---|---|---|---|---|---|---|---|---|---|---|
| | | Gama (2016) | Gama | | | | | | | | 42941 | |
| | M: Massaguet | Karal (2018) | Karal | | | | | | | | 20264 | |
| | | Massaguet | Massaguet | 62290 | 64408 | 66597 | 68862 | 71203 | 73624 | 76127 | 32935 | 2015 (A); 2016, 2017, 2018 (M) |
| | | Mani (2015) | Mani | | | | | | | | 22928 | |
| | N: Massakory | Massakory | Massakory | 28253 | 29213 | 30206 | 31233 | 32295 | 33393 | 34529 | 34529 | 2015 (A); 2016, 2017, 2018 |
| Lac | B: Bagassola | Bagassola | Bagassola | 29419 | 30419 | 31454 | 32523 | 33629 | 34772 | 35954 | 35954 | 2016, 2017, 2018 (G) |
| | E: Bol | Bol | Bol | 43409 | 44885 | 46411 | 47989 | 49621 | 51308 | 53053 | 35187 | 2013 (S); 2017, 2018 (G) |
| | | Liwa (2014) | Liwa | | | | | | | | 17865 | |
| | V: Ngouri | Isserom (2017) | Isserom | | | | | | | | 20997 | |
| | | Kouloudia (‘14) | Kouloudia | 57923 | 59892 | 61928 | 64034 | 66211 | 68462 | 70790 | 16954 | 2017, 2018 (G) |
| | | Ngouri | Ngouri | | | | | | | | 32838 | |
| Mayo Kebbi Est | F: Bongor | Bongor | Bongor | 43050 | 44514 | 46027 | 47592 | 49210 | 50883 | 52613 | 52613 | 2013 (S); 2016, 2017, 2018 (M) |
| | I: Guelendeng | Guelendeng | Guelendeng | 39968 | 41327 | 42732 | 44185 | 45687 | 47241 | 48847 | 23268 | 2017, 2018 (G) |
| | | Moulkou (‘17) | Moulkou | | | | | | | | 25579 | |
| Mayo Kebbi Ouest | J: Léré | Binder (2016) | Binder | | | | | | | | 15543 | |
| | | Guegou (2017) | Guegou | | | | | | | | 7791 | |
| | | Guelao (2017) | Guelao | 59377 | 61396 | 63483 | 65642 | 67874 | 70181 | 72568 | 9839 | 2013 (S) |
| | | Lagon (2016) | Lagon | | | | | | | | 19319 | |
| | | Léré | Léré | | | | | | | | 20076 | |
| N'Djaména | R: N'Djaména Centre | N'Djaména Centre | N'Djaména Centre | 70137 | 72521 | 74987 | 77537 | 80173 | 82899 | 85717 | 85717 | 2016, 2017, 2018 (M) |
| | S: N'Djaména Est | N'Djaména Est | N'Djaména Est | 68546 | 70876 | 73286 | 75778 | 78354 | 81018 | 83773 | 83773 | 2016, 2017, 2018 (M) |
| | T: N'Djaména Sud** | N'Djaména Sud | N'Djaména Sud | 100257 | 103666 | 107191 | 110835 | 114604 | 118500 | 122529 | 122529 | 2016, 2017, 2018 (M) |
| | U: N'Djaména Nord | N'Djaména Nord | N'Djaména Nord | 30714 | 31758 | 32838 | 33954 | 35108 | 36302 | 37536 | 37536 | 2016, 2017, 2018 (M) |

†Years of establishment of each health district created post-2013 shown in brackets. *In 2016, the 2013 district of Moussoro (Q) was subdivided into the districts of Chaddra, Michemire, Moussoro and Salal. In that year, SMC implementation took place in the districts of Moussoro and Chaddra (representing 74.5% of the population of the 2013 district), but not in Michemire and Salal. **Including the 9th arrondisement of N'Djaména. Abbreviations for organisations supporting SMC implementation: A: ACCESS-SMC (Achieving Catalytic Expansion of Seasonal Malaria Chemoprevention in the Sahel); C: French Red Cross (*Croix-Rouge française*); G: Global Fund (the Global Fund to Fight AIDS, Tuberculosis and Malaria); M: Malaria Consortium; S: Chadian state (national malaria control programme/*Programme National de Lutte contre le Paludisme du Tchad*); U: UNICEF (United Nations Children's Fund).

## Supplemental Table S2. Summary of regions of Chad and eligible health districts by year (2013, 2018 and 2019) , which did not receive SMC (2013–2018)

| Region | Health District† | | | Population projection (age 0–59 months), 2013 health districts | | | | | | | 2019 districts |
|---|---|---|---|---|---|---|---|---|---|---|---|
| 2013 | 2013 | 2018 | 2019 | 2013 | 2014 | 2015 | 2016 | 2017 | 2018 | 2019 | |
| Batha | Yao | Yao | Yao | 18716 | 19352 | 20010 | 20690 | 21394 | 22121 | 22873 | 22873 |
| Kanem | Mao | Mao | Mao | 47250 | 48856 | 50517 | 52235 | 54011 | 55847 | 57746 | 44721 |
| | | N'Tiona (2016) | N'Tiona | | | | | | | | 13025 |
| | Mondo | Mondo | Mondo | 21192 | 21912 | 22657 | 23428 | 24224 | 25048 | 25899 | 25899 |
| | Nokou | Nokou | Nokou | 3140 | 3246 | 3357 | 3471 | 3589 | 3711 | 3837 | 3837 |
| | Rig Rig | Rig Rig | Rig Rig | 11038 | 11413 | 11801 | 12202 | 12617 | 13046 | 13490 | 13490 |
| Ouaddaï | Abdi | Abdi | Abdi | 24755 | 25596 | 26467 | 27367 | 28297 | 29259 | 30254 | 30254 |
| | Abeche | Abeche | Abeche | 82824 | 85640 | 88552 | 91563 | 94676 | 97895 | 101223 | 79452 |
| | | Abougoudam (‘14) | Abougoudam | | | | | | | | 21771 |
| | Adre | Adre | Adre | 69271 | 71626 | 74062 | 76580 | 79183 | 81876 | 84659 | 84659 |
| Salamat | Aboudéia | Aboudéia | Aboudéia | 21126 | 21844 | 22587 | 23355 | 24149 | 24970 | 25819 | 25819 |
| Sila | Am-Dam | Am-Dam | Am-Dam | 19029 | 19676 | 20345 | 21037 | 21752 | 22492 | 23256 | 23256 |
| | Goz-Beida | Goz-Beida | Goz-Beida | 74472 | 77004 | 79622 | 82330 | 85129 | 88023 | 91016 | 57262 |
| | | Koukou (2015) | Koukou | | | | | | | | 33753 |
| | Tissi | Tissi | Tissi | 16234 | 16786 | 17356 | 17947 | 18557 | 19188 | 19840 | 19840 |
| Wadi Fira | Biltine | Amzoer (2017) | Amzoer | 43178 | 44646 | 46164 | 47734 | 49357 | 51035 | 52770 | 12587 |
| | | Arada (2017) | Arada | | | | | | | | 9125 |
| | | Biltine | Biltine | | | | | | | | 31058 |
| | Guereda | Guereda | Guereda | 46345 | 47921 | 49550 | 51235 | 52977 | 54778 | 56640 | 56640 |
| | Iriba | Iriba | Iriba | 34257 | 35421 | 36626 | 37871 | 39159 | 40490 | 41867 | 41867 |
| | Matadjana | Matadjana | Matadjana | 4956 | 5125 | 5299 | 5479 | 5666 | 5858 | 6057 | 6057 |

†Years of establishment of each health district created post-2013 shown in brackets.

## Supplemental Table S3. Summary of regions of Chad and health districts ineligible to receive SMC by year (2013, 2018 and 2019)

| Region | Health District† | | | Population projection (age 0–59 months), 2013 health districts | | | | | | | 2019 districts |
|---|---|---|---|---|---|---|---|---|---|---|---|
| 2013 | 2013 | 2018 | 2019 | 2013 | 2014 | 2015 | 2016 | 2017 | 2018 | 2019 | |
| Bourkou | Faya | Faya | Faya | 21746 | 22486 | 23250 | 24041 | 24858 | 25703 | 26577 | 8638 |
| | | Kirdimi (2016) | Kirdimi | | | | | | | | 7176 |
| | | Kouba Olanga (2018) | Kouba Olanga | | | | | | | | 10764 |
| Ennedi* | Amdjarass | Amdjarass | Amdjarass | 17250 | 17836 | 18443 | 19070 | 19718 | 20388 | 21082 | 9961 |
| | | Kaoura (2015) | Kaoura | | | | | | | | 7466 |
| | | Mourdi (2018) | Mourdi | | | | | | | | 3654 |
| | Bahaï | Bahaï | Bahaï | 10231 | 10579 | 10939 | 11311 | 11695 | 12093 | 12504 | 12504 |
| | Fada | Fada | Fada | 14071 | 14549 | 15044 | 15556 | 16084 | 16631 | 17197 | 5018 |
| | | | Ouniaga (2019) | | | | | | | | 6132 |
| | | Kalait (2015) | Kalait | | | | | | | | 6046 |
| Guera | Melfi | Melfi | Melfi | 22623 | 23392 | 24187 | 25010 | 25860 | 26739 | 27648 | 27648 |
| Logone Occidental | Benoye | Benoye | Benoye | 47455 | 49069 | 50737 | 52462 | 54246 | 56090 | 57997 | 57997 |
| | Laokassy | Laokassy | Laokassy | 30595 | 31635 | 32711 | 33823 | 34973 | 36162 | 37391 | 37391 |
| | Moundou | Moundou | Moundou | 92775 | 95930 | 99191 | 102564 | 106051 | 109657 | 113385 | 93927 |
| | | Baynamare (2016) | Baynamare | | | | | | | | 19458 |
| Logone Oriental | Bebedija | Bebedija | Bebedija | 45117 | 46651 | 48238 | 49878 | 51574 | 53327 | 55140 | 37480 |
| | | Donia (2017) | Donia | | | | | | | | 17660 |
| | Bessao | Bessao | Bessao | 29953 | 30971 | 32024 | 33113 | 34239 | 35403 | 36606 | 36606 |
| | Bodo | Bodo | Bodo | 23521 | 24321 | 25148 | 26003 | 26887 | 27801 | 28747 | 28747 |
| | Beboto | Beboto | Beboto | 11413 | 11801 | 12203 | 12618 | 13047 | 13490 | 13949 | 13949 |
| | Doba | Doba | Doba | 50993 | 52727 | 54519 | 56373 | 58290 | 60272 | 62321 | 34059 |
| | | | Baibokoum (2019) | | | | | | | | 15016 |
| | | Kara (2017) | Kara | | | | | | | | 13245 |
| | Gore | Gore | Gore | 26439 | 27338 | 28268 | 29229 | 30223 | 31250 | 32313 | 32313 |
| | Laramanaye | Laramanaye | Laramanaye | 11056 | 11432 | 11821 | 12223 | 12638 | 13068 | 13512 | 13512 |
| Mandoul | Goundi | Goundi | Goundi | 35716 | 36930 | 38186 | 39484 | 40826 | 42214 | 43650 | 43650 |
| | Bedjondo | Bedjondo | Bedjondo | 42898 | 44357 | 45865 | 47424 | 49037 | 50704 | 52428 | 52428 |

| | | | | | | | | | | | |
|---|---|---|---|---|---|---|---|---|---|---|---|
| | Koumra | Bedaya (2017) | Bedaya | 44374 | 45883 | 47443 | 49056 | 50724 | 52449 | 54232 | 15225 |
| | | Koumra | Koumra | | | | | | | | 39008 |
| | Moissala | Bouna (2014) | Bouna | 57030 | 58969 | 60974 | 63047 | 65190 | 67407 | 69699 | 22639 |
| | | Békourou (2018) | Békourou | | | | | | | | 11440 |
| | | Moissala | Moissala | | | | | | | | 35619 |
| Mayo Kebbi Est | Fianga | Fianga | Fianga | 52919 | 54718 | 56579 | 58502 | 60491 | 62548 | 64675 | 43324 |
| | | Youe (2016) | Youe | | | | | | | | 21350 |
| | Gounou-Gaya | Gounou-Gaya | Gounou-Gaya | 53127 | 54933 | 56801 | 58732 | 60729 | 62794 | 64929 | 44526 |
| | | Pont Carol (2016) | Pont Carol | | | | | | | | 20403 |
| Mayo Kebbi Ouest | Pala | Gagal (2016) | Gagal | 74355 | 76883 | 79497 | 82200 | 84995 | 87885 | 90873 | 26673 |
| | | Lamé (2016) | Lamé | | | | | | | | 19008 |
| | | Pala | Pala | | | | | | | | 30710 |
| | | Torrock (2016) | Torrock | | | | | | | | 14481 |
| Moyen-Chari | Danamadji | Danamadji | Danamadji | 49984 | 51684 | 53441 | 55258 | 57137 | 59079 | 61088 | 22660 |
| | | | Koumogo (2019) | | | | | | | | 16513 |
| | | Maro (2017) | Maro | | | | | | | | 21915 |
| | Kyabé | Biobé (2016) | Biobé | 39882 | 41238 | 42640 | 44090 | 45589 | 47139 | 48742 | 18528 |
| | | Kyabé | Kyabé | | | | | | | | 30214 |
| | Sarh | Korbol (2017) | Korbol | 48709 | 50366 | 52078 | 53849 | 55679 | 57573 | 59530 | 8790 |
| | | Sarh | Balimba (2019) | | | | | | | | 12228 |
| | | | Sarh | | | | | | | | 38512 |
| Salamat | Am-Timan | Am-Timan | Am-Timan | 54693 | 56553 | 58475 | 60463 | 62519 | 64645 | 66843 | 66843 |
| | Hazare-Mangue | Hazare-Mangue | Hazare-Mangue | 19299 | 19956 | 20634 | 21336 | 22061 | 22811 | 23587 | 23587 |
| Tandjile | Béré | Béré | Béré | 18331 | 18954 | 19599 | 20265 | 20954 | 21667 | 22403 | 22403 |
| | Donomanga | Donomanga | Donomanga | 24294 | 25120 | 25974 | 26857 | 27771 | 28715 | 29691 | 19588 |
| | | | Guidari (2019) | | | | | | | | 10103 |

| | | | | | | | | | | | |
|---|---|---|---|---|---|---|---|---|---|---|---|
| Tandjile | Kelo | Baktchoro (2017) | Baktchoro | 80187 | 82913 | 85732 | 88647 | 91661 | 94778 | 98000 | 19068 |
| | | Dafra (2016) | Dafra | | | | | | | | 11973 |
| | | Kelo | Kelo | | | | | | | | 52191 |
| | | | Kolon (2019) | | | | | | | | 14768 |
| | Laï | Laï | Laï | 34943 | 36131 | 37359 | 38629 | 39943 | 41301 | 42705 | 42705 |
| Tibesti | Bardaï | Bardaï | Bardaï | 5491 | 5678 | 5871 | 6071 | 6277 | 6491 | 6711 | 4811 |
| | | Zouar (2017) | Zouar | | | | | | | | 1900 |

†Years of establishment of each health district created post-2013 shown in brackets. *Ennedi Est (comprising Amdjarass and Bahaï health districts) and Ennedi Ouest (comprising Fada health district) from 2015 onwards. **Not eligible for SMC, however SMC campaigns were carried out in Moissala by Médecins Sans Frontières (MSF) France in 2015 and 2016 during July to October.
