# Supplementary material for: Modeled Impact of Seasonal Malaria Chemoprevention on District-Level Suspected and Confirmed Malaria Cases in Chad Based on Routine Clinical Data (2013–2018)
Source: Am J Trop Med Hyg. 2021 Oct 18;105(6):1712–21. doi: 10.4269/ajtmh.21-0314 (PMC8641328; doi:10.4269/ajtmh.21-0314)
Supplement: Supplementary file 2 [file tpmd210314.SD2.pdf]

Supplement Image

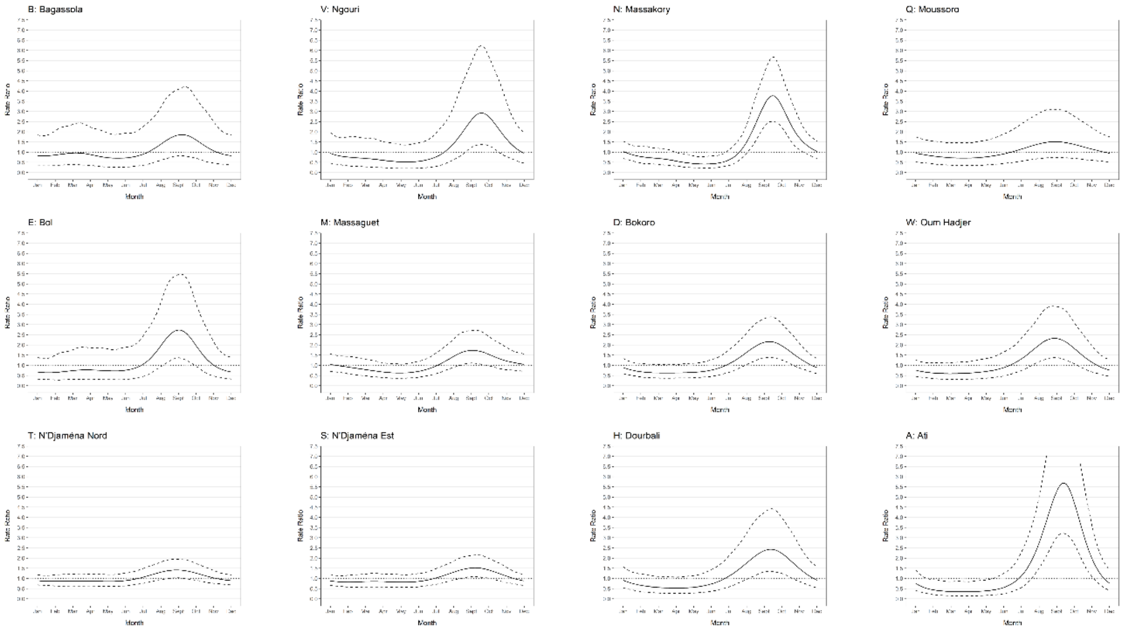

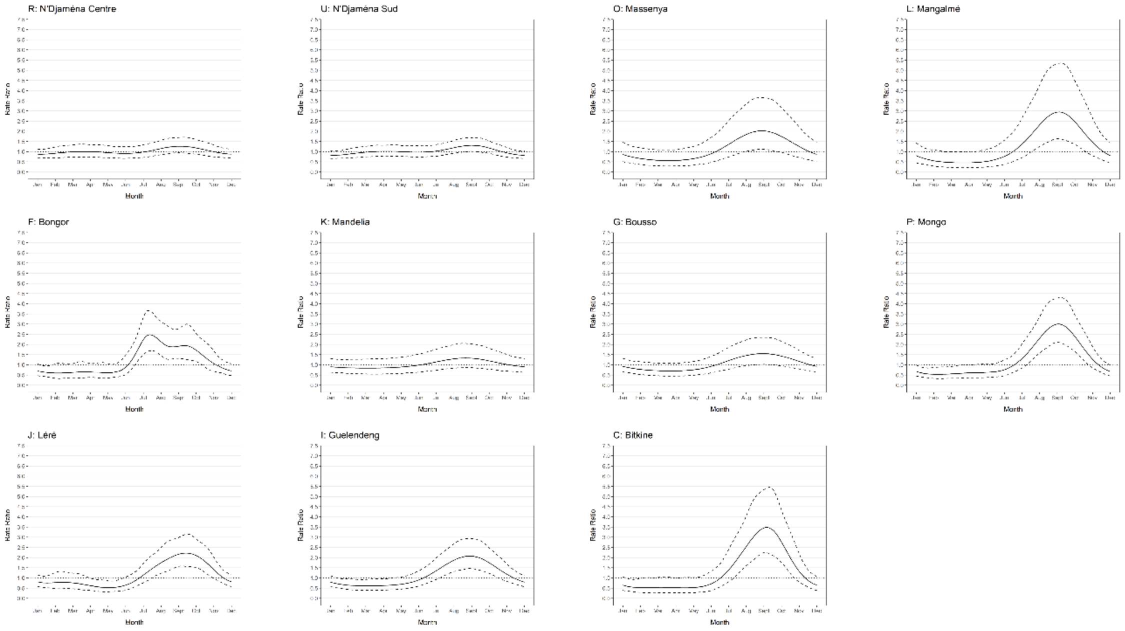

Online supplement

Supplemental Table S1. Summary of regions of Chad and eligible health districts by year (2013, 2018 and 2019) which received SMC (2013–2018)

Region	Health District†			Population projection (age 0–59 months), 2013 health districts							2019 districts	Years of SMC implementation
2013	2013	2018	2019	2013	2014	2015	2016	2017	2018	2019		
Barh El Gazel	Q: Moussoro	Chaddra (2014)	Chaddra	60012	62053	64163	66344	68600	70932	73344	18211	2016* 2017, 2018 (G)
		Michemire (2016)	Michemire								9234	
		Moussoro	Moussoro								36430	
		Salal (2016)	Salal								9469	
Batha	A: Ati	Alifa (2016)	Alifa	52750	54543	56398	58315	60298	62348	64468	23219	2015 (C)
		Ati	Ati								12564	
			Djedda (2019)								11139	
			Koundjourou (2019)								17546	
	W: Oum-Hadjer	Assinet (2016)	Assinet	41745	43165	44632	46150	47719	49341	51019	19539	2015 (C)
		Oum-Hadjer	Oum-Hadjer								31480	
Chari Baguirmi	G: Bousso	Bailli (2016)	Bailli	49257	50932	52664	54454	56306	58220	60200	26876	2016, 2017, 2018 (M)
		Bousso	Bousso								24376	
		Kouno (2016)	Kouno								8947	
	H: Dourbali	Dourbali	Dourbali	30899	31949	33036	34159	35320	36521	37763	37763	2015 (A); 2016, 2017, 2018 (M)
	K: Mandelia	Mandelia	Mandelia	29966	30985	32039	33128	34254	35419	36623	36623	2015 (A); 2016, 2017, 2018 (M)
	O: Massenya	Massenya	Massenya	29062	30050	31071	32128	33220	34350	35518	35518	2015 (A); 2016, 2017, 2018 (M)
Guera	C: Bitkine	Bitkine	Bitkine	39527	40871	42261	43698	45184	46720	48308	48308	2015, 2016, 2018 (U)
	L: Mangalamé	Mangalamé	Mangalamé	22223	22979	23760	24568	25403	26267	27160	27160	2014, 2015, 2016, 2018 (U)
	P: Mongo	Baro (2016)	Baro	40476	41852	43275	44747	46268	47841	49468	12939	2013 (S); 2015, 2016, 2018 (U)
		Mongo	Mongo								36529	
Hadjer Lamis	D: Bokoro	Bokoro	Bokoro	81102	83859	86711	89659	92707	95859	99118	56178	2017, 2018 (G)

		Gama (2016)	Gama								42941	
	M: Massaguet	Karal (2018)	Karal	62290	64408	66597	68862	71203	73624	76127	20264	2015 (A); 2016, 2017, 2018 (M)
		Massaguet	Massaguet								32935	
		Mani (2015)	Mani								22928	
	N: Massakory	Massakory	Massakory	28253	29213	30206	31233	32295	33393	34529	34529	2015 (A); 2016, 2017, 2018
Lac	B: Bagassola	Bagassola	Bagassola	29419	30419	31454	32523	33629	34772	35954	35954	2016, 2017, 2018 (G)
	E: Bol	Bol	Bol	43409	44885	46411	47989	49621	51308	53053	35187	2013 (S); 2017, 2018 (G)
		Liwa (2014)	Liwa								17865	
	V: Ngouri	Isserom (2017)	Isserom	57923	59892	61928	64034	66211	68462	70790	20997	2017, 2018 (G)
		Kouloudia ('14)	Kouloudia								16954	
		Ngouri	Ngouri								32838	
Mayo Kebbi Est	F: Bongor	Bongor	Bongor	43050	44514	46027	47592	49210	50883	52613	52613	2013 (S); 2016, 2017, 2018 (M)
	I: Guelendeng	Guelendeng	Guelendeng	39968	41327	42732	44185	45687	47241	48847	23268	2017, 2018 (G)
		Moulkou ('17)	Moulkou								25579	
Mayo Kebbi Ouest	J: Léré	Binder (2016)	Binder	59377	61396	63483	65642	67874	70181	72568	15543	2013 (S)
		Guegou (2017)	Guegou								7791	
		Guelao (2017)	Guelao								9839	
		Lagon (2016)	Lagon								19319	
		Léré	Léré								20076	
N'Djaména	R: N'Djaména Centre	N'Djaména Centre	N'Djaména Centre	70137	72521	74987	77537	80173	82899	85717	85717	2016, 2017, 2018 (M)
	S: N'Djaména Est	N'Djaména Est	N'Djaména Est	68546	70876	73286	75778	78354	81018	83773	83773	2016, 2017, 2018 (M)
	T: N'Djaména Sud**	N'Djaména Sud	N'Djaména Sud	100257	103666	107191	110835	114604	118500	122529	122529	2016, 2017, 2018 (M)
	U: N'Djaména Nord	N'Djaména Nord	N'Djaména Nord	30714	31758	32838	33954	35108	36302	37536	37536	2016, 2017, 2018 (M)

†Years of establishment of each health district created post-2013 shown in brackets. *In 2016, the 2013 district of Moussoro (Q) was subdivided into the districts of Chaddra, Michemire, Moussoro and Salal. In that year, SMC implementation took place in the districts of Moussoro and Chaddra (representing 74.5% of the population of the 2013 district), but not in Michemire and Salal. **Including the 9th arrondisement of N'Djaména. Abbreviations for organisations supporting SMC implementation: A: ACCESS-SMC (Achieving Catalytic Expansion of Seasonal Malaria Chemoprevention in the Sahel); C: French Red Cross (*Croix-Rouge française*); G: Global Fund (the Global Fund to Fight AIDS, Tuberculosis and Malaria); M: Malaria Consortium; S: Chadian state (national malaria control programme/*Programme National de Lutte contre le Paludisme du Tchad*); U: UNICEF (United Nations Children's Fund).

Supplemental Table S2. Summary of regions of Chad and eligible health districts by year (2013, 2018 and 2019) , which did not receive SMC (2013–2018)

Region	Health District†			Population projection (age 0–59 months), 2013 health districts							2019 districts
2013	2013	2018	2019	2013	2014	2015	2016	2017	2018	2019	
Batha	Yao	Yao	Yao	18716	19352	20010	20690	21394	22121	22873	22873
Kanem	Mao	Mao<br>N'Tiona (2016)	Mao<br>N'Tiona	47250	48856	50517	52235	54011	55847	57746	44721<br>13025
	Mondo	Mondo	Mondo	21192	21912	22657	23428	24224	25048	25899	25899
	Nokou	Nokou	Nokou	3140	3246	3357	3471	3589	3711	3837	3837
	Rig Rig	Rig Rig	Rig Rig	11038	11413	11801	12202	12617	13046	13490	13490
Ouaddaï	Abdi	Abdi	Abdi	24755	25596	26467	27367	28297	29259	30254	30254
	Abeche	Abeche<br>Abougoudam (‘14)	Abeche<br>Abougoudam	82824	85640	88552	91563	94676	97895	101223	79452<br>21771
	Adre	Adre	Adre	69271	71626	74062	76580	79183	81876	84659	84659
Salamat	Aboudéia	Aboudéia	Aboudéia	21126	21844	22587	23355	24149	24970	25819	25819
Sila	Am-Dam	Am-Dam	Am-Dam	19029	19676	20345	21037	21752	22492	23256	23256
	Goz-Beida	Goz-Beida<br>Koukou (2015)	Goz-Beida<br>Koukou	74472	77004	79622	82330	85129	88023	91016	57262<br>33753
	Tissi	Tissi	Tissi	16234	16786	17356	17947	18557	19188	19840	19840
Wadi Fira	Biltine	Amzoer (2017)<br>Arada (2017)<br>Biltine	Amzoer<br>Arada<br>Biltine	43178	44646	46164	47734	49357	51035	52770	12587<br>9125<br>31058
	Guereda	Guereda	Guereda	46345	47921	49550	51235	52977	54778	56640	56640
	Iriba	Iriba	Iriba	34257	35421	36626	37871	39159	40490	41867	41867
	Matadjana	Matadjana	Matadjana	4956	5125	5299	5479	5666	5858	6057	6057

†Years of establishment of each health district created post-2013 shown in brackets.

Supplemental Table S3. Summary of regions of Chad and health districts ineligible to receive SMC by year (2013, 2018 and 2019)

Region	Health District†			Population projection (age 0–59 months), 2013 health districts							2019 districts
2013	2013	2018	2019	2013	2014	2015	2016	2017	2018	2019	
Bourkou	Faya	Faya	Faya	21746	22486	23250	24041	24858	25703	26577	8638
		Kirdimi (2016)	Kirdimi								7176
		Kouba Olanga (2018)	Kouba Olanga								10764
Ennedi*	Amdjarass	Amdjarass	Amdjarass	17250	17836	18443	19070	19718	20388	21082	9961
		Kaoura (2015)	Kaoura								7466
		Mourdi (2018)	Mourdi								3654
	Bahaï	Bahaï	Bahaï	10231	10579	10939	11311	11695	12093	12504	12504
	Fada	Fada	Fada	14071	14549	15044	15556	16084	16631	17197	5018
			Ouniaga (2019)								6132
		Kalait (2015)	Kalait								6046
Guera	Melfi	Melfi	Melfi	22623	23392	24187	25010	25860	26739	27648	27648
Logone Occidental	Benoye	Benoye	Benoye	47455	49069	50737	52462	54246	56090	57997	57997
	Laokassy	Laokassy	Laokassy	30595	31635	32711	33823	34973	36162	37391	37391
	Moundou	Moundou	Moundou	92775	95930	99191	102564	106051	109657	113385	93927
		Baynamare (2016)	Baynamare								19458
Logone Oriental	Bebedija	Bebedija	Bebedija	45117	46651	48238	49878	51574	53327	55140	37480
		Donia (2017)	Donia								17660
	Bessao	Bessao	Bessao	29953	30971	32024	33113	34239	35403	36606	36606
	Bodo	Bodo	Bodo	23521	24321	25148	26003	26887	27801	28747	28747
	Beboto	Beboto	Beboto	11413	11801	12203	12618	13047	13490	13949	13949
	Doba	Doba	Doba	50993	52727	54519	56373	58290	60272	62321	34059
			Baibokoum (2019)								15016
		Kara (2017)	Kara								13245
	Gore	Gore	Gore	26439	27338	28268	29229	30223	31250	32313	32313
	Laramanaye	Laramanaye	Laramanaye	11056	11432	11821	12223	12638	13068	13512	13512
Mandoul	Goundi	Goundi	Goundi	35716	36930	38186	39484	40826	42214	43650	43650
	Bedjondo	Bedjondo	Bedjondo	42898	44357	45865	47424	49037	50704	52428	52428

	Koumra	Bedaya (2017)	Bedaya	44374	45883	47443	49056	50724	52449	54232	15225
		Koumra	Koumra								39008
	Moissala	Bouna (2014)	Bouna	57030	58969	60974	63047	65190	67407	69699	22639
		Békourou (2018)	Békourou								11440
		Moissala	Moissala								35619
Mayo Kebbi Est	Fianga	Fianga	Fianga	52919	54718	56579	58502	60491	62548	64675	43324
		Youe (2016)	Youe								21350
	Gounou-Gaya	Gounou-Gaya	Gounou-Gaya	53127	54933	56801	58732	60729	62794	64929	44526
		Pont Carol (2016)	Pont Carol								20403
Mayo Kebbi Ouest	Pala	Gagal (2016)	Gagal	74355	76883	79497	82200	84995	87885	90873	26673
		Lamé (2016)	Lamé								19008
		Pala	Pala								30710
		Torrock (2016)	Torrock								14481
Moyen-Chari	Danamadji	Danamadji	Danamadji	49984	51684	53441	55258	57137	59079	61088	22660
			Koumogo (2019)								16513
		Maro (2017)	Maro								21915
	Kyabé	Biobé (2016)	Biobé	39882	41238	42640	44090	45589	47139	48742	18528
		Kyabé	Kyabé								30214
	Sarh	Korbol (2017)	Korbol	48709	50366	52078	53849	55679	57573	59530	8790
		Sarh	Balimba (2019)								12228
			Sarh								38512
Salamat	Am-Timan	Am-Timan	Am-Timan	54693	56553	58475	60463	62519	64645	66843	66843
	Hazare-Mangue	Hazare-Mangue	Hazare-Mangue	19299	19956	20634	21336	22061	22811	23587	23587
Tandjile	Béré	Béré	Béré	18331	18954	19599	20265	20954	21667	22403	22403
	Donomanga	Donomanga	Donomanga	24294	25120	25974	26857	27771	28715	29691	19588
			Guidari (2019)								10103

Tandjile	Kelo	Baktchoro (2017)	Baktchoro	80187	82913	85732	88647	91661	94778	98000	19068
		Dafra (2016)	Dafra								11973
		Kelo	Kelo								52191
			Kolon (2019)								14768
	Laï	Laï	Laï	34943	36131	37359	38629	39943	41301	42705	42705
Tibesti	Bardaï	Bardaï	Bardaï	5491	5678	5871	6071	6277	6491	6711	4811
		Zouar (2017)	Zouar								1900

†Years of establishment of each health district created post-2013 shown in brackets. *Ennedi Est (comprising Amdjarass and Bahaï health districts) and Ennedi Ouest (comprising Fada health district) from 2015 onwards. **Not eligible for SMC, however SMC campaigns were carried out in Moissala by Médecins Sans Frontières (MSF) France in 2015 and 2016 during July to October.
